# Influence of key histological characteristics on 18F-fluorodeoxyglucose /18F-choline positron emission tomography positivity in hepatocellular carcinoma: A machine learning study

**DOI:** 10.3389/fmed.2023.1087957

**Published:** 2023-01-19

**Authors:** Jérôme Ghidaglia, Vincent Laurent, Mylène Sebagh, Alina Pascale, Emmanuel Durand, Nicolas Golse, Florent L. Besson

**Affiliations:** ^1^Department of Biophysics and Nuclear Medicine-Molecular Imaging, Hôpitaux Universitaires Paris-Saclay, Assistance Publique-Hôpitaux de Paris, Le Kremlin-Bicêtre, France; ^2^Université Paris-Saclay, Centre Borelli, Gif-sur-Yvette, France; ^3^Department of Pathology, Hôpitaux Universitaires Paris-Saclay, Assistance Publique-Hôpitaux de Paris, Le Kremlin-Bicêtre, France; ^4^Universite Paris-Saclay, Inserm, Physiopathogènése et Traitement des Maladies du Foie, UMR-S 1193, Villejuif, Île-de-France, France; ^5^Université Paris-Saclay, School of Medicine, Le Kremlin-Bicêtre, France; ^6^Centre Hépato Biliaire, Hepatobiliary and Liver Transplant Unit, Hôpitaux Universitaires Paris-Saclay, Assistance Publique-Hôpitaux de Paris, Villejuif, France; ^7^Université Paris-Saclay, Commissariat à l’énergie atomique et aux énergies alternatives (CEA), Centre National de la Recherche Scientifique (CNRS), Inserm, BioMaps, Le Kremlin-Bicêtre, France

**Keywords:** hepatocellular carcinoma, PET/CT, 18F-FDG, 18F-choline, machine learning

## Abstract

**Purpose:**

To determine the characteristics influence of key histological on 18F-fluorodeoxyglucose (18F-FDG) and 18F-choline positron emission tomography (PET) positivity in hepatocellular carcinoma (HCC).

**Materials and methods:**

The 18F-FDG/18F-choline PET imaging findings of 103 histologically proven HCCs (from 62 patients, of which 47 underwent hepatectomy and 15 received liver transplantation) were retrospectively examined to assess the following key histological parameters: Grade, capsule, microvascular invasion (mVI), macrovascular invasion (MVI), and necrosis. Using a ratio of 70/30 for training and testing sets, respectively, a penalized classification model (Elastic Net) was trained using 100 repeated cross-validation procedures (10-fold cross-validation for hyperparameter optimization). The contribution of each histological parameter to the PET positivity was determined using the Shapley Additive Explanations method. Receiver operating characteristic curves with and without dimensionality reduction were finally estimated and compared.

**Results:**

Among the five key histological characteristics of HCC (Grade, capsule, mVI, MVI, and necrosis), mVI and tumor Grade (I–III) showed the highest relevance and robustness in explaining HCC uptake of 18F-FDG and 18F-choline. MVI and necrosis status both showed high instability in outcome predictions. Tumor capsule had a minimal influence on the model predictions. On retaining only mVI and Grades I–III for the final analysis, the area under the receiver operating characteristic (ROC) curve values were maintained (0.68 vs. 0.63, 0.65 vs. 0.64, and 0.65 vs. 0.64 for 18F-FDG, 18F-choline, and their combination, respectively).

**Conclusion:**

18F-FDG/18F-choline PET positivity appears driven by both the Grade and mVI components in HCC. Consideration of the tumor microenvironment will likely be necessary to improve our understanding of multitracer PET positivity.

## Introduction

Hepatocellular carcinoma (HCC) is a leading cause of cancer death worldwide, and thus represents a major health challenge (1). While conventional imaging remains essential for the management of HCC (2–5), positron emission tomography (PET) imaging has been increasingly used in this field (6, 7). In particular, 18F-fluorodeoxyglucose (18F-FDG) and 18F-choline PET radiotracers, which, respectively target the carbohydrate and fatty acid metabolic pathways, have shown their complementarity in clinical practice for the initial staging and treatment management of HCC (8–11). At present, combined use of 18F-FDG and 18F-choline is still not standard practice, partially because their biological significance in HCC remains largely unknown. Seminal papers have suggested that there is an inverse PET behavior in terms of their uptake in HCC, according to their grade of differentiation; namely, poorly-differentiated tumors tend to be 18F-FDG-avid, whereas well-differentiated tumors tend to be choline-avid (12, 13). Although attractive, these historical findings have been recently challenged by the results of our recent dedicated literature synthesis based on 99 HCCs reported over the past 16 years (14). To date, little is known about the true influence of key histological characteristics of HCC on 18F-FDG/18F-choline PET uptake.

Following our systematic review (14), we conducted the present original study to determine the influence of key histological characteristics on 18F-FDG and 18F-choline PET positivity in HCC.

## Materials and methods

The present study was conducted in compliance with the tenets of the declaration of Helsinki. According to the rules of our institution, all patients were systematically informed of data collection and research purposes. All included patients were specifically informed and did not object to this study, which was approved by our University Ethical Committee (IRB no. CEPS-440).

### Database characteristics

A total of 103 consecutive histologically proven HCCs (62 patients, of whom 47 were treated by hepatectomy and 15 by liver transplantation) with available baseline 18F-FDG and 18F-choline PET data were retrospectively reviewed. For all patients, the whole surgical sample was available, and the following histological characteristics were reported for each HCC tumor: degree of differentiation [Grade I, II, or III of the WHO classification (15)]; presence or absence of a capsule; microvascular invasion (mVI): microscopic neoplastic emboli discovered on histology; macrovascular invasion (MVI): macroscopic emboli visible with the naked eye during management of the macroscopic part and/or visible through imaging; and presence or absence of necrosis. The 18F-FDG and 18F-choline PET data were acquired before surgery on the same hybrid PET/computed tomography (CT) device (Biograph mCT FlowMotion, Siemens Healthineers, Erlangen, Germany), and were reconstructed using the same 3D iterative algorithm (3D time-of-flight–ordered subset expectation maximization method, two iterations and 21 subsets with time-of-flight and point spread function modeling, and with random, dead time, scatter, decay, and attenuation corrections; matrix size = 400 × 400), with postfiltering (Gaussian filter: 3.0 mm).

### 18F-FDG and 18F-choline image analyses

All HCC PET data were reviewed by two nuclear imaging physicians (JG and FLB, with 4 and 12 years of expertise in hybrid PET imaging, respectively) on the same professional workstation (syngo.via, Siemens Healthineers, Munich, Germany). For both 18F-FDG and 18F-choline, any focal radiotracer uptake compared to the surrounding liver background was considered positive, whereas iso or hypometabolic lesions were both considered negative. Any disagreements between the two readers were resolved by consensus. Additionally, for each HCC tumor, the peak standardized uptake values normalized by the lean body mass (SUL_peak_) were semi-automatically extracted. The choice of SULpeak was motivated by technical considerations of robustness and reliability (16, 17).

### Statistical analyses

All categorical data are expressed as numbers and percentages, while continuous data are expressed as medians and interquartile ranges. A machine learning structured procedure was performed lesion-wise to decipher the influence of all HCC histological features on 18F-FDG and 18F-choline dual-tracer PET uptake behaviors, and to select the most relevant ones. This procedure was as follows:

(1) Univariate correlation analyses (Spearman’s non-parametric rank correlations) were performed to identify monotonic relationships between binarized histological characteristics (Grade, capsule, necrosis, mVI, and MVI) and dual-radiotracer visual PET uptake behaviors (positive for 18F-FDG, 18F-choline, or both). To note, because Grade II–an intermediate with potential overlaps between very well-differentiated (Grade I) and poorly differentiated (Grade III) tumors–was under represented, and to avoid any linear relationship between the variables during the label encoding process, only the Grades I and III were encoded, while capturing the entire information of the dataset.

(2) For each of the 18F-FDG, 18F-choline, and dual-PET tracer behaviors, a general linear regression classifier (L1 + L2 regularization, ElasticNet) was used to assess the impact of HCC histological components on PET tracer positivity. The overall dataset was split into training and test sets at a 70–30% ratio. To prevent potential overfitting and measure model stability, the learning procedures were 10-fold cross-validated for hyperparameter optimization (the lesions of a patient belong to the same sample), and the final models were repeated 100 times. For the three PET behaviors, the contribution of each histological component to the model’s prediction (PET positivity uptake) was deciphered using the Shapley Additive Explanations (SHAP) method, and the performances of the models were assessed by estimating the corresponding receiver operating characteristic (ROC) curves.

(3) Finally, a feature selection process was performed by fine-tuning the regularization path of the regression models (grid search: α ranged from 0.1 to 100, and the L1/L2 ratio ranged from 0 to 1), and the ultimate penalization coefficients were chosen based on model’s stability, simplicity, and overall performance. ROC curves of the dimensionality-reduced models (including the most relevant histological features) were estimated and compared to the non-dimensionality-reduced models.

All statistical analyses were performed using Python (version 3.8.8; Python Software Foundation)^1^ and Scikit-Learn library (version 1.1)^2^. For all analyses, statistical significance was set at *p* < 0.05.

## Results

### Database characteristics

**FIGURE 1:**
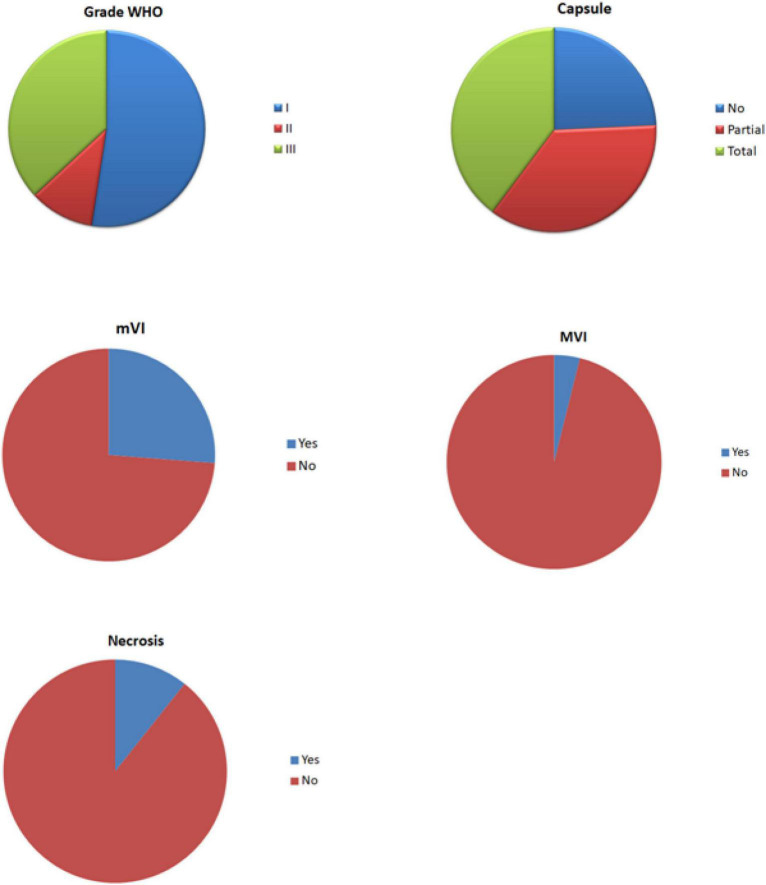
Pie chart of the histological characteristics of hepatocellular carcinomas (HCCs).

The characteristics of all included patients, together with histological characteristics of their HCCs, are shown in Figure 1 and Table 1. Briefly, the 62 included patients had a mean age of 66 years (range: 56–76 years), and 55 (89%) were male. Liver disease was explicitly reported for 55 patients (89%), and 47 were treated by surgery (76%) and 15 by liver transplantation (24%). Each patient had a mean of 1.5 (range: 1–2) HCCs, and the mean delay between the two PET/CT examinations was 5.5 (range: 4–7) days. At the lesion level (*n* = 103), a total of 54, 11, and 38 tumors were classified as Grade I (52%), II (11%), and III (37%), respectively. The presence of a capsule was reported in 78 cases (76%), and vascular invasion was reported for 31 tumors.

**TABLE 1:** Dataset characteristics.

Patient characteristics (*n* = 62)	
Sex (M/F)	55/7
Age in years	66 (56–76)
Treatment procedure	
● Surgery	*n* = 47
● Liver transplantation	*n* = 15
Liver disease	
● No evident liver disease	*n* = 7
● Viral infection (HCV, HBV)	*n* = 22
● Non-alcoholic steatohepatitis (NASH)/ethylism (OH)	*n* = 20
● Other	*n* = 14
AFP	6.25 (3–10.5)
Number of lesions per patient	1.5 (1–2)
BCLC classification	
● A	*n* = 44
● B	*n* = 10
● C	*n* = 0
● D	*n* = 2
● Unclassified	*n* = 6
Delay between 18F-FDG and 18F-choline imaging (days)	5.5 (4–7)
Patient’s blood glucose (mmol/L)	5.5 (4–7)
Weight (kg)	77.5 (70–85)
Injected dose 18F-FDG (MBq)	271.5 (241–302)
Delay between injection and PET (FDG) (min)	60 (60–60)
Injected dose of 18F-choline (MBq)	161.5 (136–187)
Delay between injection and PET (choline) (min)	25 (20–30)
**Lesion characteristics (*n* = 103)**	
Grade WHO	
● I	*n* = 54
● II	*n* = 11
● III	*n* = 38
Capsule	
● No	*n* = 25
● Partial	*n* = 37
● Total	*n* = 41
Vascular invasion	
● Macro	*n* = 4
● Micro	*n* = 27
Necrosis	
● Yes	*n* = 11
● No	*n* = 92

### 18F-FDG and 18F-choline image analyses

**FIGURE 2:**
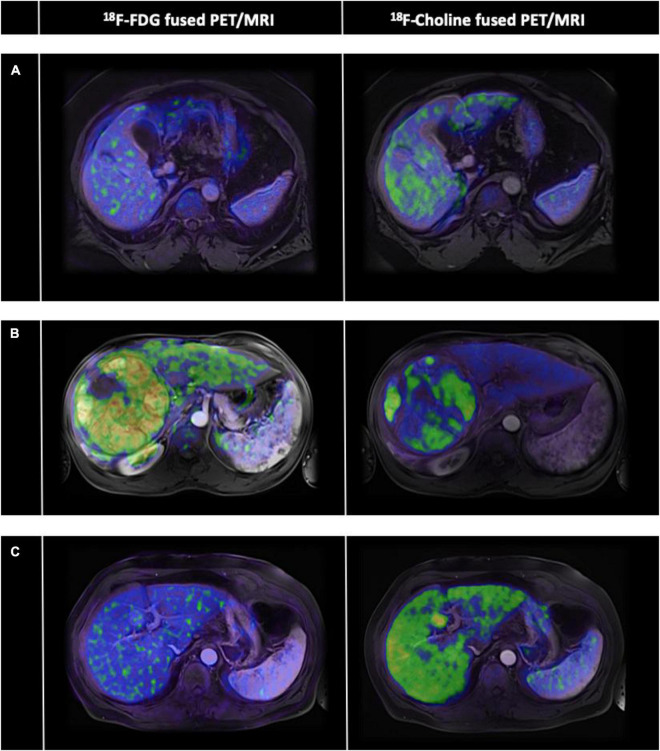
Case-mix of hepatocellular carcinoma (HCC) with various dual-tracer positron emission tomography (PET) profiles. The PET part of the non-contrast-enhanced PET/computed tomography (CT) data 18F fluorodeoxyglucose (18F-FDG on the left panel and 18F-choline on the right panel) of three HCCs **(A–C)** were retrospectively fused to their available contrast-enhanced MRI data (axial T1-weighted MRI pulse sequences with arterial phase contrast enhancement). **(A)** Dual-PET radiotracers negative uptake (HCC Grade III, with mVI). **(B)** Dual-PET radiotracers positive uptake (HCC Grade I, with mVI). **(C)** Negative 18F-FDG uptake but positive 18F-choline uptake (HCC Grade I-II, without mVI).

**FIGURE 3:**
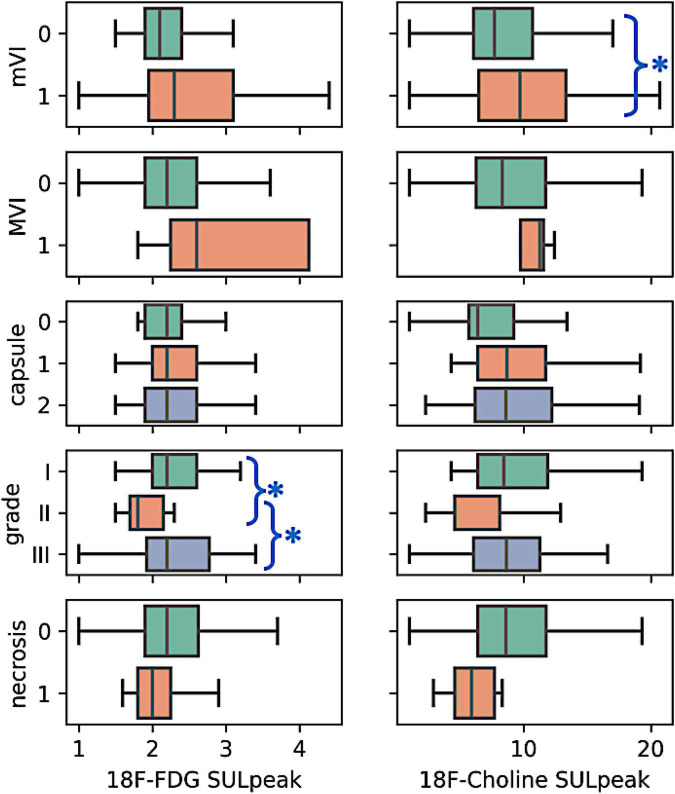
Boxplot of the semi quantitative positron emission tomography (PET) data according to histological characteristics. Among the five histological characteristics, higher 18F- choline uptake was observed in the case of mVI positive tumors (*p* < 0.05, non-parametric mann-whitney U test, blue asterisk). Also, 18F-FDG uptake between the Grades I–II and II–III was significant (*p* < 0.05, non-parametric kruskal wallis ANOVA with *post-hoc* tests, blue asterisk). For the remaining macrovascular invasion (MVI), capsule (no, partial, total), and necrosis (absence, presence) characteristics, no difference in radiotracers uptake was observed between the tumor subgroups (*p* > 0.05).

The visual analyses of the PET imaging data are summarized in Table 2, upper panel. Briefly, 56% of the HCC tumors negative for 18F-FDG were Grade I tumors, whereas 55% of HCC tumors positive for 18F-FDG were Grade III tumors. On the other hand, 62% of the HCC tumors positive for 18F-choline were Grade I tumors, and up to 36% of them were Grade III. On total, 35 HCC tumors (34% of the whole dataset, 20 Grade I and 15 Grade III) were positive for both the two radiotracers. Concerning the capsule status, its presence was observed in 73% of the 18F-FDG PET positive HCC cases and 77% of the 18F-FDG PET negative HCC cases, while 78% of the 18F-choline PET positive and 74% of the 18F-choline PET negative HCC cases showed evidence of capsule. For vascular invasion, macro vascular invasion was reported in 9% of the 18F-FDG PET positive HCC cases and 1% of the 18F-FDG PET negative HCC cases, and in 7 and 2% of HCC cases for 18F-choline PET positive and 18F-choline PET negative cases, respectively. Finally, mVI was reported in 42% of the 18F-FDG PET positive HCC cases versus 19% for the 18F-FDG PET negative HCC cases, and 38% of the 18F-choline PET positive HCC cases versus 17% for the 18F-choline PET negative HCC cases. HCCs with the various dual-tracer PET behaviors are shown in Figure 2. As illustrated in Figure 3, the intensity of 18F-FDG and 18F-choline PET uptakes assessed using SUL_peak_ showed higher radiotracer concentration in cases of mVI (18F-choline only) and between the Grade I–II and II–III (for 18F-FDG only). There were no other between-group differences.

**TABLE 2:** Positron emission tomography (PET) results.

HCC (*n* = 103)	Visual analysis			
	18F-FDG		18F-choline	
	Positive	Negative	Positive	Negative
Grade WHO				
● I	15/33 (45%)	39/70 (56%)	28/45 (62%)	26/58 (45%)
● II	0/33 (0%)	11/70 (16%)	1/45 (2%)	10/58 (17%)
● III	18/33 (55%)	20/70 (29%)	16/45 (36%)	22/58 (38%)
Capsule				
● No	9/33 (27%)	16/70 (23%)	10/45 (22%)	15/58 (26%)
● Partial	13/33 (39%)	24/70 (34%)	18/45 (40%)	19/58 (33%)
● Total	11/33 (33%)	30/70 (43%)	17/45 (38%)	24/58 (41%)
Vascular invasion				
● Macro	3/33 (9%)	1/70 (1%)	3/45 (7%)	1/58 (2%)
● Micro	14/33 (42%)	13/70 (19%)	17/45 (38%)	10/58 (17%)
**HCC (*n* = 103)**	**Semi-quantitative analysis**			
	**18F-FDG**		**18F-choline**	
Grade WHO				
● I	3.0 (2–4)		6.5 (4–9)	
● II	4.25 (1–7.5)		5.5 (3–8)	
● III	3.0 (2–4)		6.5 (4–9)	
Capsule				
● No	3.0 (2–4)		6.0 (4–8)	
● Partial	4.0 (2–6)		6.5 (4–9)	
● Total	3.0 (1–5)		6.5 (4–9)	
Vascular invasion				
● Macro	4.75 (1.5–8)		7.25 (3.5–11)	
● Micro	4.0 (2–6)		6.5 (4–9)	

For the upper panel of the table, the ratios represent the number of tumors verifying the histological characteristic of interest normalized by the positron emission tomography (PET) status. The related percentages are also provided.

### Contribution of key histological characteristics on 18F-FDG and 18F-choline PET positivity

**FIGURE 4:**
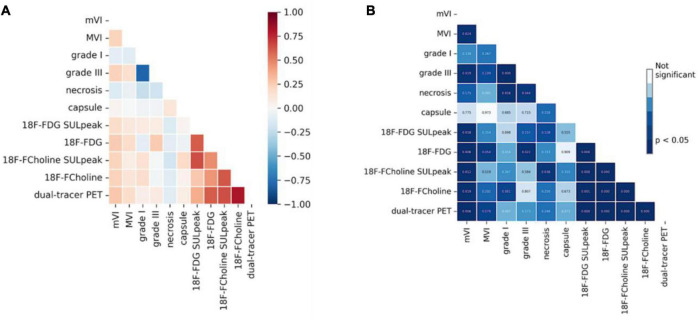
Heatmap of the correlations between histological and positron emission tomography (PET) metrics (Spearman’s rank correlation). **(A)** Spearman ρcoefficients between histological characteristics of hepatocellular carcinoma (HCC) and PET uptake positivity. **(B)** Corresponding *p*-values Here, only the mVI and Grade III were significantly correlated to the PET metrics.

**FIGURE 5:**
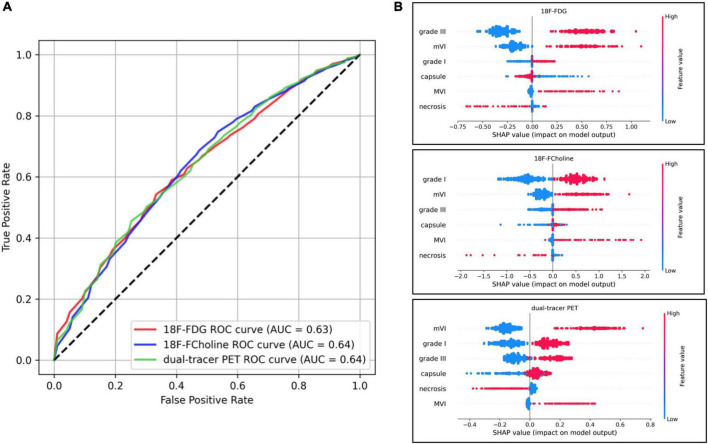
Model performances including all histological parameters, and Shapley Additive Explanations (SHAP) plot analyses. **(A)** Receiver operating characteristic (ROC) curves of the three models’ performances. The ROC curve illustrates the capacity of classifiers to discriminate positive and negative cases at different thresholds (e.g., for the 18F-fluorodeoxyglucose (18F-FDG) model and a desired 60% positive rate, the algorithm will yield 40% false alarms). The random model is represented by identity (dotted line) and the perfect model (with no randomness) is *y* = 1 on [0, 1]. **(B)** Impact on positron emission tomography (PET) positivity is shown with SHAP values on the x-axis, and values of histological characteristics are shown in color (blue and red for low and high values, respectively).

**FIGURE 6:**
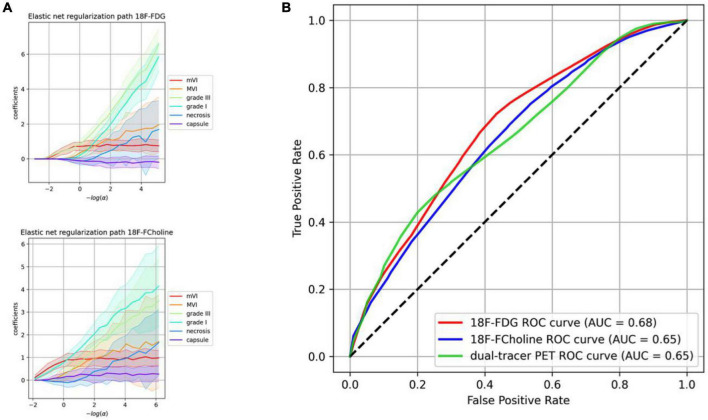
Regularization paths and model performances including the most relevant histological parameters finally retained. **(A)** Regularization paths for 18F-fluorodeoxyglucose (18F-FDG) and 18F-choline. The more the penalization decreases (right side), the more the coefficients are scattered. **(B)** Receiver operating characteristic (ROC) curves of the dimensionality-reduced models’ performance. The ROC curve illustrates the capacity of classifiers to discriminate positive and negative cases at different thresholds (e.g., for the 18F-FDG model and a desired 60% positive rate, the algorithm will yield 38% of false alarms). The random model is represented by identity (dotted line), and the perfect model (with no randomness) is *y* = 1 on [0, 1].

A correlation heatmap showing the relationships between the histological characteristics of the 103 HCCs and their PET behaviors is provided in Figure 4. The three PET uptake behaviors were significantly correlated with mVI (18F-FDG positivity: Spearman’s ρ = 0.25, *p* = 0.01; 18F-choline positivity: ρ = 0.23, *p* = 0.02; 18F-FDG/18F-choline positivity: ρ = 0.27, *p* = 0.006). A significant correlation between 18F-FDG PET positivity and tumor differentiation (Grade III) was also observed (ρ = 0.25, *p* = 0.01). No other histological–PET correlations were significant. Considering all the histological characteristics (Grade, capsule, necrosis, mVI, and MVI), the regression classifier provided area under the ROC curve (AUC) values of 0.63, 0.64, and 0.64 for 18F-FDG, 18F-choline, and 18F-FDG/18F-choline PET positivity, respectively [Figure 5A (G)]. As shown in Figure 5B (G), Grade I–III and mVI histological parameters contributed most to explaining PET positivity in all three cases (18F-FDG, 18F-choline, and dual-tracer positivity), whereas capsule, necrosis, and MVI parameters were non-relevant. Figure 6A (H) shows the impact of the regularization paths on coefficient scattering. Based on their stability, which emphasizes their interpretability [Figure 5B (G)], the mVI and Grades I–III were retained in the final analysis. By fixing the regularization parameter to 0.5, AUC values of the dimensionality-reduced models were maintained [Figure 6B (H)].

## Discussion

In this study, which included data from 103 histologically proven HCCs, a structured machine learning procedure was used to ascertain the influence of the main histological characteristics of HCC on 18F-FDG and 18F-choline PET uptake. Among the five key histological characteristics (Grade, capsule, mVI, MVI, and necrosis), mVI and tumor Grade (I–III) showed the highest relevance and robustness in explaining HCC uptake of 18F-FDG and 18F-choline. MVI and necrosis status both showed high instability in outcome predictions, which highlights their non-relevance in model building. Tumor capsule had a very weak influence on model predictions.

This is the first study to specifically examine the influence of several key histological characteristics of HCC on 18F-FDG/18F-choline PET positivity, and our results complement previous findings on PET imaging of HCC. First, our recent systematic review, which included 99 HCCs from six studies, revealed there to be a very large overlap of 18F-FDG/18F-choline PET findings between well- and less-differentiated histological subtypes (14). While Grade III was significantly correlated with 18F-FDG PET visual positivity in the present study, no difference in SUL_peak_ was observed between Grade I and III for this radiotracer. Second, we found significant correlations of both 18F-FDG and 18F-choline positivity with microvascular invasion (mVI). To note, HCCs can be broadly classified as proliferative or non-proliferative tumors according to their level of differentiation, genetic and epigenetic features, and immunological characteristics (18). Based on this emerging molecular classification, the proliferative class includes poorly differentiated tumors with a high degree of vascular invasion, while the non-proliferative class corresponds to well-to-moderately differentiated tumors with less vascular invasion. Furthermore, a significant relationship between 18F-FDG uptake and mVI has been reported previously (19–21). Our rigorous feature selection process, which was performed without *a priori*, showed that among the common key histological features, Grade and mVI were the most relevant parameters in explaining the 18F-FDG and 18F-choline PET positivity in HCCs. In accordance with findings of seminal studies (12, 13), our results suggest that 18F-FDG/18F-choline dual-tracer PET positivity is driven by the Grade (poorly differentiated for 18F-FDG and well-differentiated for 18F choline), but also microvascular invasion, rather than the Grade alone. Moreover, we found that these key histological characteristics of HCC only moderately predicted PET positivity (AUCs of 68, 65, and 65% for 18F-FDG, 18F-choline, and 18F-FDG/18F-choline, respectively, vs. an AUC of 50% in the random model). As in any tumor, HCC cells are surrounded by a complex cellular microenvironment with close interactions (21, 22). In such an ecosystem, immune cell infiltrate appears to be critical for tumor growth and adaptability (23, 24), and could even be a defining feature of an emerging subclasses of tumor phenotypes (18, 23). To note, high immune activity has been observed in both non-proliferative and proliferative HCCs (18). Thus, in all these tumors, the 18F-FDG and 18F-choline PET signatures are also likely to be partially associated with increased immune activity (24, 25). Based on these considerations, and given the fact that all grade II tumors exhibited low avidity both for 18F-FDG and 18F-choline in our study, new molecular radioprobes would be of particular interest to improve the clinical relevance of PET imaging in the management of HCC. PET radiotracers targeting the immune microenvironment of tumor cells (26) or HCC neovasculature (27, 28) appear promising in this field.

Our study has several limitations that should be noted. First, this was a retrospective monocentric study. However, the dataset included more than 100 HCCs, for which complete histological and dual-radiotracer PET lesion data were available. Second, we applied a general linear classifier to predict PET positivity of HCCs. Moreover, we tested other machine learning procedures (gradient boosting, SVC), but these models did not converge due to the intrinsic properties of this real-life dataset. Third, we did not consider the influence of tumor microenvironment, and only focused on the most widely used histological characteristics of HCC tumor cells. Based on our results and the emerging concepts of immune-mediated phenotypes in HCC, further studies are warranted to improve our understanding of the factors underlying multitracer PET positivity in this field.

To conclude, among five widely used histological parameters, we found that Grade and microvascular invasion were the most relevant parameters in explaining both 18F-FDG and 18F-choline PET positivity in HCC. Consideration of the tumor microenvironment in future work could improve our understanding of multitracer PET positivity.

## Data availability statement

The original contributions presented in this study are included in this article/supplementary material, further inquiries can be directed to the corresponding author.

## Ethics statement

The studies involving human participants were reviewed and approved by IRB no. CEPS-440. The patients/participants provided their written informed consent to participate in this study.

## Author contributions

All authors contributed to design and acquisition analysis, revising for intellectual content, final approval, agreed to be accountable for all aspects of this work (accuracy and integrity of any part of the work), and approved the submitted version.

## Footnotes

1
http://www.python.org

2
https://scikit-learn.org
